# Supplementary material for: Myeloid - derived suppressor cells in Type 1 diabetes are an expanded population exhibiting diverse T-cell suppressor mechanisms
Source: PLoS One. 2020 Nov 18;15(11):e0242092. doi: 10.1371/journal.pone.0242092 (PMC7673497; doi:10.1371/journal.pone.0242092)
Supplement: S1 Fig — (PDF) [file pone.0242092.s001.pdf]

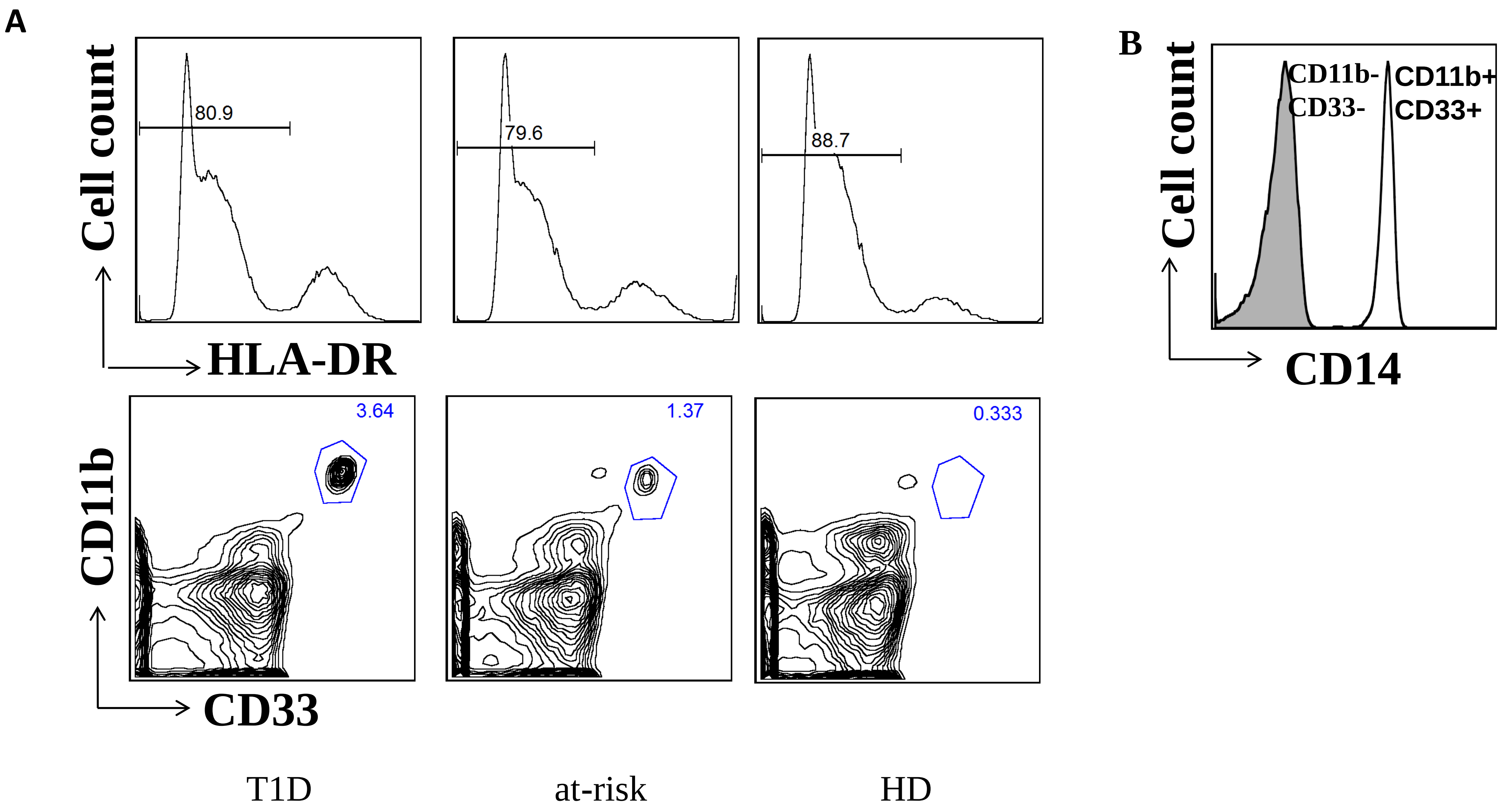

S1 Fig. The gating strategy of M-MDSC. (A) Flow cytometry plots representing gating strategy of M-MDSC ($CD11b^+CD33^+$cells) gated on $HLADR^{neg/low}$ cells from the PBMC of T1D patient, their at-risk relatives and healthy donors (HD). (B) Representative flow plot of CD14 expression in $CD11b^+$ $CD33^+$ compared to $CD11b^-CD33^-$ cells.
